# The tooth movement efficiency of different orthodontic thermoplastics for clear aligners: study protocol for a randomized controlled clinical trial

**DOI:** 10.1186/s13063-023-07736-9

**Published:** 2023-10-23

**Authors:** Chuangchuang Mu, Bingjing Sun, Zhicheng Gong, Yuanyuan Wei, Li Chen, Wei Zhang, Haimiao Wu, Bingjiao Zhao

**Affiliations:** 1 Department of Orthodontics, Shanghai Stomatological Hospital, Fudan University, No.356 Beijing East Road, Shanghai, China; 2 Department of Dental Technology, Shanghai Stomatological Hospital, Fudan University, Shanghai, China; 3 Department of Biostatistics, School of Public Health, Fudan University, Shanghai, China

**Keywords:** Orthodontic thermoplastics, Orthodontic tooth movement (OTM), Clear aligners, Randomized controlled trial

## Abstract

**Introduction:**

With regard to the esthetics and comfort of orthodontic treatment, the requirement for removable clear aligners (CAs) is increasing. Unlike conventional fixed orthodontic appliances, CAs were made of thermoplastic film by thermoforming on the personalized dental models. The construction of orthodontic thermoplastic is a critical factor for orthodontic tooth movement (OTM). Polyethylene terephthalate glycol-modified (PETG) and thermoplastic polyurethane (TPU) are the most commonly orthodontic thermoplastics; however, the evidence of the differences between different orthodontic thermoplastic are limited to vitro environment and the evidence in vivo environment is not available. Therefore, this trial aims to provide reliable evidence for orthodontists’ personalized treatment plans whether the two most commonly used orthodontic thermoplastics of PETG and TPU have differences in the efficiency of OTM.

**Methods and analysis:**

This randomized controlled clinical study will recruit 44 orthodontic patients for orthodontic treatment. All the subjects will be randomized into two groups (PETG and TPU, *n* = 22 for each group). In the first stage (M0 to M1), clear aligners will be made of two orthodontic thermoplastics and move the maxillary first or second premolars 2 mm. In the second stage, patients will take the standard orthodontic treatments. The primary outcome will be the efficiency of clear aligners made of different materials on the digital models. The secondary outcome will be the efficiency of clear aligners made of different materials on the cone-beam computed tomography (CBCT). The efficiency will be calculated through the superimposition of the digital models and CBCT.

**Discussion:**

The results from this trial will serve as evidence for orthodontists and manufacturers and clarify whether the difference in orthodontic thermoplastics significantly impacts the efficiency of OTM.

**Trial registration number:**

ChiCTR2300070980. Registered on 27 April 2023.

https://www.chictr.org.cn/showproj.html?proj=186253

## Introduction

Malocclusion can lead to negative psychological and social impacts on public health as one of the most common oral diseases [1]. Fixed appliances are the traditional treatment for malocclusion, but the requirement for personalized clear aligners made of orthodontic thermoplastics is increasing with regard to the esthetics and comfort of orthodontic treatment. The programmed movement with clear aligners provides a new method to measure increment and related factors of the orthodontic tooth movement (OTM). Several clinical researchers have studied the related factors using clear aligners. McGorray et al. [2] compared relaxin and a placebo using programmed aligners with regard to tooth movement and stability in a randomized clinical trial. They measured the movement weekly by digitized models and followed for four weeks to assess relapse. The treatment group has no differences in tooth movement in 8 and 12 weeks. Chisari et al. [3] examined the factors of age, sex, root length, bone levels, and bone quality on OTM by clear aligners. Digital models were measured for tooth movement, and cone-beam computed tomography was used to assess the bone and morphometric features. Their results show that the relationship between age and tooth movement is complex, and gender might have an influence. Nada et al. [4] studied 38 patients treated with Invisalign to compare the amount of tooth movement in predicted and achieved. The digital models were superimposed using Compare software, and the mean accuracy was 50%. The distal movement of the maxillary first premolar (57.1%) and the mesial movement of the maxillary second premolar (64.7%) were relatively accurate movements, respectively.

Apart from the clinical factors above, the construction and thickness of orthodontic thermoplastic materials are also related to the efficiency of clear aligners. Align Technology (San Jose, Calif)’s first marketed aligners were made from a single-layer rigid polyurethane and iteration by Exceed-30 and SmartTrack (a thermoplastic polyurethane, TPU) subsequently [5]. Another commonly orthodontic thermoplastics material of aligners manufactures is polyethylene terephthalate glycol-modified (PETG). Other orthodontic thermoplastic materials such as polypropylene (PP), polycarbonate (PC), and ethylene vinyl acetate (EVA) are also used [6]. The metals in fixed orthodontics have elastic properties. The deflection and tooth movement is predictable when forces are exerted by orthodontists [7, 8]. Orthodontic thermoplastic materials are viscoelastic and will occur stress relaxation in the oral cavity with intermittent loads [4, 9]. Therefore, the accurate assessment of the efficiency of aligners with different materials will increase the predictability of orthodontic treatment. Several studies have tested the aligners’ mechanical properties of different materials in vitro [10–12] environment. However, these studies are limited to observation time and vitro environment. Bollen et al. [13] compared two materials provided by Align Technology (hard and soft) and activation frequencies (1 and 2 weeks) on fifty-one subjects. They found that hard and soft aligners show no difference in the completion rate, and the frequency of 2 weeks has a higher completion rate. But the material constructions are not mentioned in their study. The tooth movement efficiency of different orthodontic thermoplastics still lacks clinical trial evidence.

Are there differences in the composition of different materials? This study will first provide reliable clinical evidence for orthodontists whether the two most commonly used orthodontic thermoplastics of PETG and TPU have differences in the efficiency of OTM. The efficiency of OTM will be evaluated by the movements of the teeth with programmed aligners.

## Methods and analysis

### Trial design

**Fig. 1:**
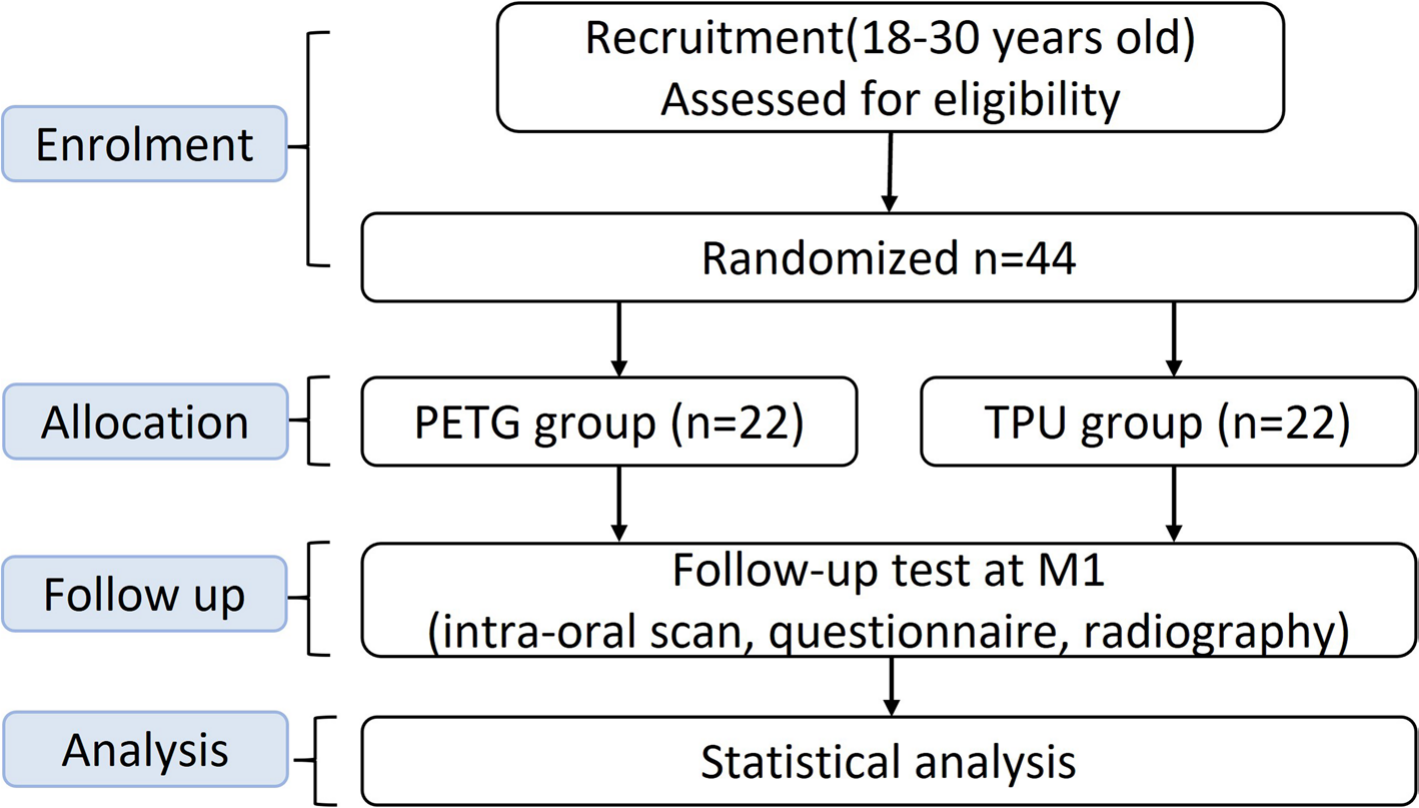
Flow chart of the trial design

This study is a parallel-group, randomized controlled, double-blinded clinical trial (Fig. 1). The protocol is following the CONSORT guidelines (2010) [14]. This trial will recruit orthodontic patients from the Department of Orthodontics, Shanghai Stomatological Hospital. Clinical research coordinators will receive training on the design, methodology, clinical intervention, and keeping trial documentation of this study first. And then, they will explain the purpose and the arrangement of this study to the patients. The patients who are willing to participate in this study will be entered into the electronic data capture (EDC) system held on the computer server of Shanghai Stomatological Hospital.

**Fig. 2:**
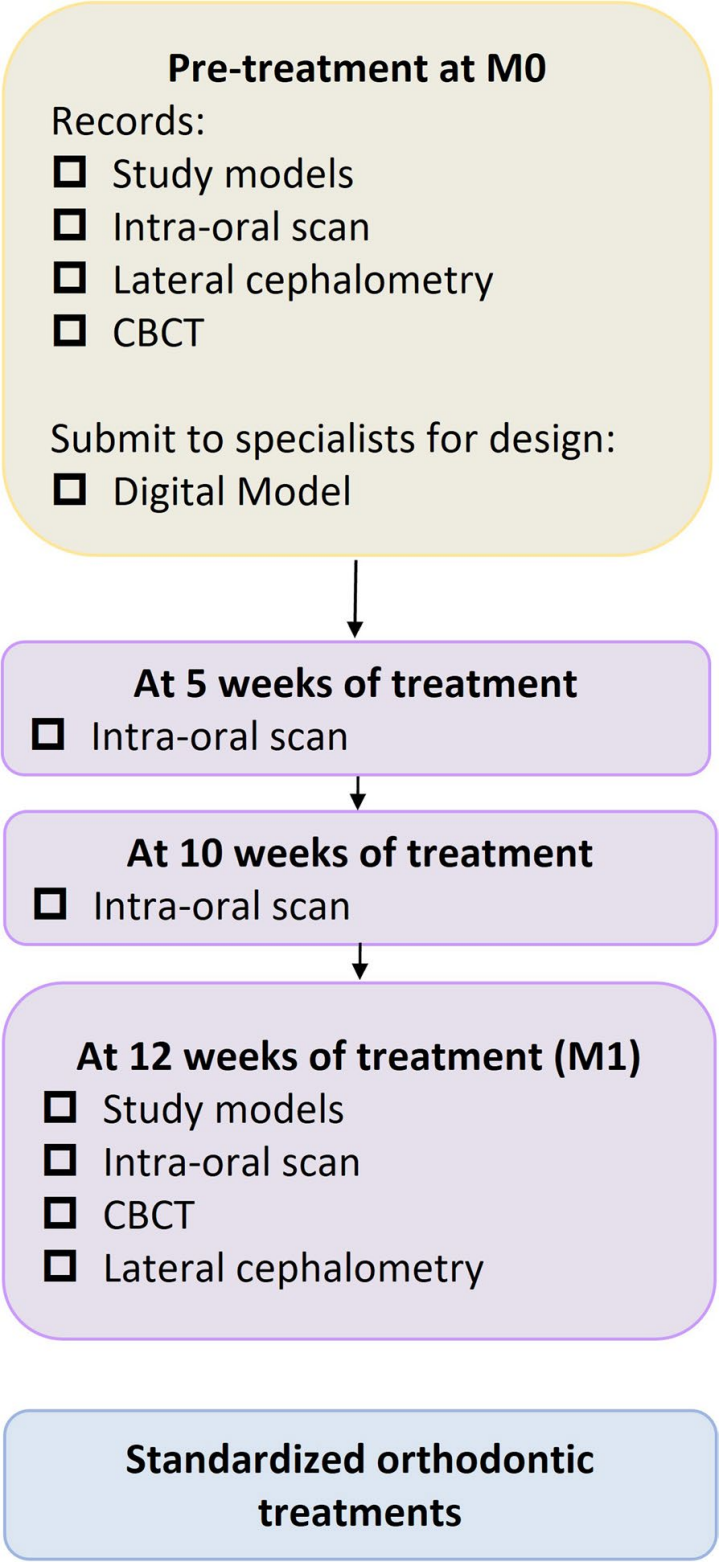
The critical nodes of this study

Each participating patient will undergo the necessary medical examinations which include physical examinations, lateral cephalometry, cone beam computed tomography (CBCT), and intra-oral scan. The participants will be randomly divided into two equal groups (PETG and TPU)—at a ratio of 1:1. Different tests of two groups (PETG and TPU) will be recorded before the treatment (M0), 12 weeks after treatment (M1). The procedure shows in Fig. 2. This is a randomized clinical trial consisting of two parallel groups with equal randomization to detect the superiority of one intervention over the other in sagittal OTM of premolars.

### Eligibility criteria

The patients will be assessed by orthodontists who have experience with clear aligners.

#### Inclusion criteria

The participants must meet the following criteria:

1. Adult patients, aged 18–40 years.
2. Permanent dentition, the first molar of the upper and lower and the anterior teeth are not missing.
3. The crowding of dentition ≥ 5 mm, orthodontic treatment requires the extraction of the maxillary first premolar or the maxillary second premolar.
4. Patients who are able to wear aligners as required time.

#### Exclusion criteria

The exclusion criteria of this study are as follows:

1. Allergic to orthodontic thermoplastics.
2. Patients need to have orthognathic (jaw) surgery.
3. With symptoms or signs of temporomandibular joint disorders.
4. With systemic diseases.

### Interventions

All participants’ maxillary first or second premolars will be selected at M0 to ensure that selected teeth can move towards the mesial or distal side 2 mm and not be blocked out by adjacent teeth. A series of 10 maxillary clear aligners were programmed to accomplish the movements from M0 to M1, and the increments of movement of the maxillary premolar will be designed as 0.2 mm in each step. The type of orthodontic thermoplastic for aligners manufactured depends on the card of their envelopes, PETG (Scheu, Iserlohn, Germany) or TPU (Maxflex, Taiwan). The clear aligners will be collected at the end of the fifth and tenth weeks. The patients will take an intra-oral scan at the same time. Routine follow-up will be conducted 2 weeks after programmed 10 steps.

The process and critical nodes of interventions in this study were showed in Fig. 2. The flow charts were printed and delivered to clinical doctors for reminders. In the first stage, from M0 to M1, the programmed movements were designed under the discussion of specialists.

Implementing use of PETG or TPU will not require alteration to usual care pathways, and the digital model registering, the aligners receiving, and the check of study number will continue for both trial arms.

### Patient and public involvement

The patients and the public will not be involved in the design, conduct, reporting, or dissemination plans in this trial.

### Outcome measures

The records of patients will be anonymized before analysis to eliminate analyst bias.

#### Primary outcome

**Fig. 3:**
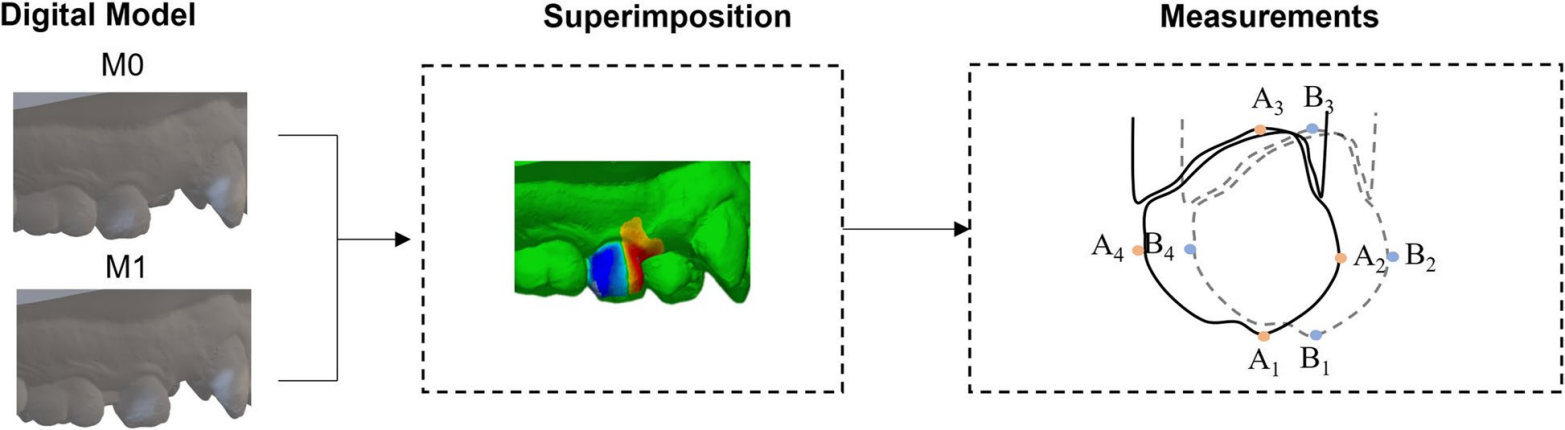
The measurements of OTM on digital models

The primary outcome will be the efficiency of clear aligners on OTM. Intra-oral scan (iTero, Align Technology, San Jose, Calif) will be collected in the key steps as shown in Fig. 2. The intra-scan digital models of different time points will be superimposed in the Rapidform2006 (INUS Technology Inc., Seoul, Korea) for measurements (Fig. 3). Landmarks’ three-dimensional coordinates in the buccal side (A1-A4, B1-B4) of the maxillary premolars will be recorded to calculate the crowns’ movements, including mesial tipping, distal tipping, buccal inclination, lingual inclination, rotation, intrusion, and extrusion.

#### Secondary outcomes

**Fig. 4:**
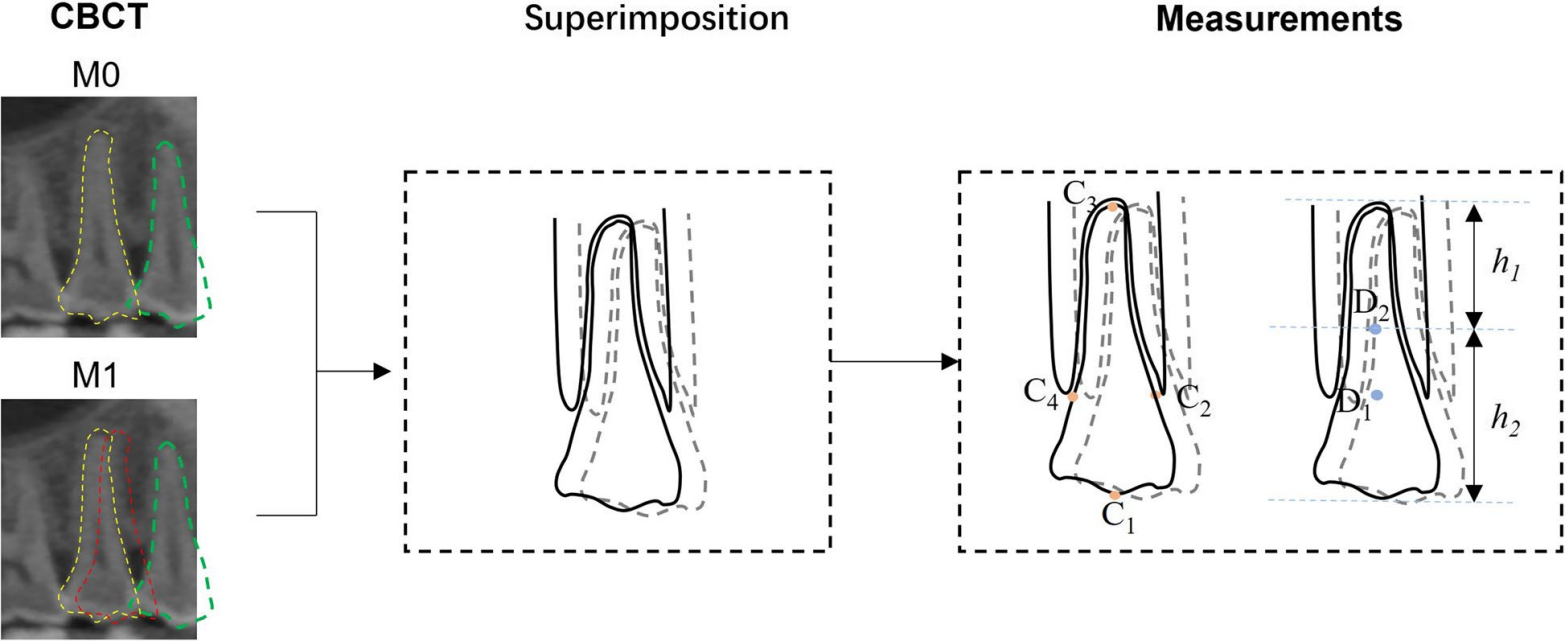
The measurements of OTM on CBCT. The planned extracted tooth is surrounded by the green dotted line. The planned moved tooth is surrounded by the yellow dotted line. The target position of the moved tooth is surrounded by the red dotted line

The CBCT images of different time points will be saved as Digital Imaging and Communications in Medicine (DICOM) format files and imported into Dolphin software (Dolphin Imaging & Management Solutions Corporation, Chatsworth, CA, USA) for superimposition based on voxel (Fig. 3.). The roots’ movements and the rotation angle will be inferred through the point marked (C_1_-C_4_, D_1_-D_2_). The lengths of the roots and crowns will also be measured (*h*_*1*_*, h*_*2*_) (Fig. 4). The efficiency will finally be calculated with the ratio of clinical tooth movements and designed movements. Correlations between age and OTM, as well as between gender and OTM, will be analyzed.

### Target sample size

The sample size was calculated using PASS software (Utah, USA) and based on the primary outcome in this trial. The expectation hypothesis is that TPU orthodontic thermoplastics will increase the efficacy of orthodontic tooth movement (OTM). The studies of Chisari JR [3] and McGorray SP et al. [2] were used to estimate standard deviation. The standard deviation of OTM is 0.8 (TPU) and 0.616 (PETG) in their study. The sample size of 20 patients in a single group will achieve 80% power to detect a difference in 10 weeks with a 0.05 two-sided significance level. Considering a 10% dropout rate, this trial will recruit a total of 44 patients.

### Participant recruitment

Patients will be recruited at the orthodontic department in Shanghai Stomatological Hospital from 1 June 2023 to 31 December 2024 (anticipated). The notification will be published on the official website and hospital. The patients will be assessed by their orthodontists whether meet the inclusion criteria. The patients invited will have at least 2 weeks to make a decision.

### Sequence generation and randomization

Block randomization was used in this study. The subjects who met the criteria were randomly assigned to the experimental group and the control group according to the ratio of 1:1. A random allocation table was generated by an independent statistician through SAS 9.4 (SAS Institute Inc., Cary, NC, USA) software. Randomization allocation and masking were achieved by the envelope method.

### Participant allocation

In this study, patients’ allocation will be accomplished by numbered, opaque, sealed envelopes. The envelopes will be kept by the dental technician in charge of aligner manufacture. After the orthodontic design is confirmed, the dental technician will open the allocation envelope to identify the type of orthodontic thermoplastics. The allocation envelope will only be opened when clear aligners need to be manufactured.

### Blinding

**Fig. 5:**
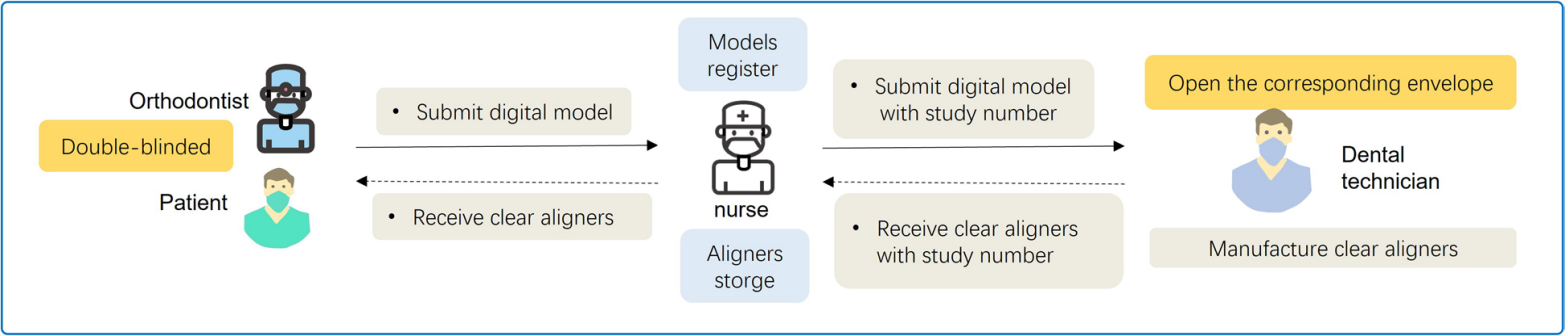
The diagram of double-blinded design

This study is a double-blinded clinical trial (Fig. 5). The nurse collects the digital model registered with a study number and submits it to the dental technicians. The dental technicians will know and keep the group allocation when the corresponding envelope is open before manufacture. Then, unaware of the group allocation, the nurse receives the clear aligners with the study number. Because of the similar transparency and elasticity, clear aligners made of PETG and TPU are hard to distinguish. Thus, the orthodontists and patients only know the study number and are blinded for group allocation. The group allocation and patients’ information will be blinded to the analyst at the analyzing stage.

### Data collection and management

#### Plans for data and records collected

The data will be recorded in case report forms (CRFs) designed by specialists and Zhejiang Taimei Technology Co., Ltd. The CRF will be filled out for each patient by coordinators. The fixed period of supervision will be taken by a qualified clinical research assistant. The clinical research assistant is blinded to the group allocation and reviews this trial’s informed consent and CRFs. The original CRFs will be saved in the data management center of Shanghai Stomatological Hospital.

### Data management

All patients’ image data, questionnaires, and measurements will be collected and checked regularly. The primary data will be coded to allow for the blinding of the analyst. The coded data will be exported into SPSS 24.0 (Chicago, the USA) for further analysis when the trial is accomplished.

### Post-trial care

All files of this trial will be retained for at least ten years at the end of the study.

### Confidentiality

All patients’ data will be securely stored with limited access. All participants will be informed that their records will have the possibility to be reviewed by the sponsor, the research ethics committee (EC), and the regulatory authority. All unpublished results and records will be deemed confidential. Any published reports will not contain personal data for the protection of personal privacy.

### *Statistical* methods

#### Statistical methods for outcomes

The primary and secondary outcomes are continuous variables. Descriptive statistics will be first used to summarize the outcomes of the study and show the distribution, mean differences, and standard deviations of the data. Spearman correlation was used to test for correlation between age/gender and the efficiency of OTM. The statistical analysis of the efficiency will be performed through the one-way ANOVA test. It will be considered significant when *P* < 0.05.

### Methods to handle missing data

The multiple imputation (MI) method will be used to deal with missing data.

### Oversight and monitoring

Trial monitoring committee

The trial monitoring committee will consist of three experienced orthodontists. The committee will meet every 4 weeks to ensure the trial is being carried out under good clinical practice (GCP). All adverse events from M0 to M1 will be recorded and reported to the committee. The committee will assess and manage the adverse events. Patients have the right to quit the study or defer to the committee. The committee will decide whether the patient needs to leave when adverse events occur. The status of the leaved patient will be followed up.

### Plans for communicating important protocol amendments

Any revisions during the trial need to be reported to the research ethics committee and take permission. The informed consent will be rewritten in accordance with the modifications and re-signed with patients.

### Dissemination and publication

The data of the current study will be analyzed and performed a summary report. The final report will be published in orthodontic journals by the principal researcher.

## Discussion

The OTM is a series of complex processes of biochemical reactions, and clear aligners have been widely used for orthodontic treatment because of their esthetics and comfort [15–17]. Thus, the clinical evidence of related factors for OTM by clear aligners is critical. The previous studies mainly focus on the clinical elements of OTM using the aligners provided by companies. Few clinical studies paid attention to orthodontic thermoplastics. This study compared two commonly orthodontic thermoplastics for OTM on the maxillary central incisors. The comfort questionnaire of two materials is also involved for evaluation.

Although the features and design of the attachment for aligners will also influence the orthodontic treatment, specialists discuss and move tooth limited to maxillary premolars to avoid it. Another limitation is that double-layer and multi-layer composite materials were also taken to the market recently [12]. In addition to these two representative materials, the efficiency of composite materials still needs compare in the future.

The results from this trial will serve as evidence for orthodontists and manufacturers and clarify whether the difference in orthodontic thermoplastics significantly impacts the efficiency of OTM.

### Trial status

The study has been ongoing since 1 May 2023. The participant recruitment was started on n 1 June 2023. Recruitment is expected to be completed by the end of 31 December 2024. The protocol version is 1.0 (issue date 8 December 2022).

## Data Availability

The datasets used and/or analyzed during the current study are available from the corresponding author on reasonable request.

## Abbreviations

OTM: Orthodontic tooth movement
CAs: Clear aligners
PETG: Polyethylene terephthalate glycol-modified
TPU: Thermoplastic polyurethane
PP: Polypropylene
PC: Polycarbonate
EVA: Ethylene vinyl acetate
EDC: Electronic data capture
CBCT: Cone beam computed tomography
CRF: Case report forms
EC: Ethics committee
MI: Multiple imputation
GCP: Good clinical practice
